# EMPLOYMENT OUTCOMES AMONG OLDER ADULTS WITH A HISTORY OF INCARCERATION

**DOI:** 10.1093/geroni/igae098.0972

**Published:** 2024-12-31

**Authors:** Rodlescia Sneed

**Affiliations:** Wayne State University, Detroit, Michigan, United States

## Abstract

Older adults returning to community settings after incarceration face a number of challenges, including food insecurity, difficulty finding housing, and adverse health outcomes. A number of qualitative studies have suggested poor employment outcomes among formerly incarcerated older adults; however, there have been few quantitative inquiries related to employment outcomes for these older adults. The purpose of this study was to understand associations between history of incarceration and employment outcomes among older adults. We used pooled data from the 2012 and 2014 waves of the Health and Retirement Study to examine associations between history of incarceration and employment outcomes among community-dwelling older adults aged >50 (n=4,985). History of incarceration and employment outcomes were assessed via self-report. While there were no associations between history of incarceration and employment status in the sample, employed older adults with a history of incarceration had more physically demanding jobs and reported more work stress and more work-related discrimination than their counterparts. They also reported less work satisfaction and less supervisor support than never-incarcerated older adults. Gender moderated the association between history of incarceration and several employment outcomes, with associations observed among women but not men. Taken together, our findings suggest that history of incarceration is associated with significant differences in employment outcomes, primarily among women. Future studies should explore factors contributing to poor employment outcomes in order to design interventions that improve outcomes in this population.

